# Lifestyle, Diet, and Colorectal Cancer Risk According to (Epi)genetic Instability: Current Evidence and Future Directions of Molecular Pathological Epidemiology

**DOI:** 10.1007/s11888-017-0395-0

**Published:** 2017-12-02

**Authors:** Laura A. E. Hughes, Colinda C. J. M. Simons, Piet A. van den Brandt, Manon van Engeland, Matty P. Weijenberg

**Affiliations:** 1 Department of Epidemiology, GROW School for Oncology and Developmental Biology, Maastricht University, Maastricht, the Netherlands; 2 Department of Pathology, GROW School for Oncology and Developmental Biology, Maastricht University Medical Centre, Maastricht, the Netherlands

**Keywords:** Colorectal cancer, Molecular pathological epidemiology, Diet, Lifestyle, Review

## Abstract

**Purpose of Review:**

In this review, we describe molecular pathological epidemiology (MPE) studies from around the world that have studied diet and/or lifestyle factors in relation to molecular markers of (epi)genetic pathways in colorectal cancer (CRC), and explore future perspectives in this realm of research. The main focus of this review is diet and lifestyle factors for which there is evidence for an association with CRC as identified by the World Cancer Research Fund reports. In addition, we review promising hypotheses, that warrant consideration in future studies.

**Recent Findings:**

Associations between molecular characteristics of CRC have been published in relation to smoking, alcohol consumption; body mass index (BMI); waist:hip ratio; adult attained height; physical activity; early life energy restriction; dietary acrylamide, fiber, fat, methyl donors, omega 3 fatty acids; meat, including total protein, processed meat, and heme iron; and fruit and vegetable intake.

**Summary:**

MPE studies help identify where associations between diet, lifestyle, and CRC risk may otherwise be masked and also shed light on how timing of exposure can influence etiology. Sample size is often an issue, but this may be addressed in the future by pooling data.

## Introduction

Colorectal cancer (CRC) is the third most common cancer in the world, regardless of sex, with nearly 1.4 million cases diagnosed in 2012 [1]. The majority of these cancers (70–80%) are sporadic in nature [2], and if current trends continue, it is estimated that 2.2 million cases of CRC will be diagnosed annually worldwide by 2030 [1]. It is now well accepted that CRC risk is highly modifiable through diet and lifestyle; recent reports suggest that up to 47% of CRC cases could be prevented by staying physically active, maintaining a healthy body weight and eating a healthy diet [3].

The expert panel of the World Cancer Research Fund (WCRF), which is the organization responsible for publishing the most comprehensive review to date on risk factors related to diet and physical activity for cancer, has recently concluded that there is convincing strong evidence that body fatness, adult attained height, and consuming processed meat and alcoholic drinks increase the risk of developing CRC, while physical activity decreases the risk of developing CRC. Furthermore, they concluded that consuming whole grains, foods containing dietary fiber, dairy products and calcium supplements probably protect against CRC, and consuming red meat probably increases the risk of developing CRC [3].

CRC is not a single disease, but rather encompasses a heterogeneous complex of diseases characterized by numerous genetic and epigenetic abnormalities [4•]. Recently, several studies have used unsupervised clustering methods to develop genomic signatures to classify colorectal cancer (CRC) into different subtypes, and have shown that each subtype has distinct molecular features and prognosis [5•]. As summarized by Song et al. [5•], the CRC Assigner (CRCA) classifier categorized CRC into 5 distinct subtypes: enterocyte, gobletlike, inflammatory, stemlike, and transit amplifying (TA) [6]; and the Colon Cancer Subtypes (CCS) classifier identified 3 groups: CCS1, CCS2, and CCS3 [7]. Several studies have shown that different classifiers are highly correlated; for example, for CCS and CRCA classifiers, most CCS1 tumors are classified as TA or enterocyte, most CCS2 tumors are classified as inflammatory and gobletlike tumors, and most CCS3 tumors are classified as stemlike tumors [8•, 9]. Although these classifications may be significant in the advancement of CRC research, these subtypes will not be specifically addressed in this review, as they have not yet been investigated in MPE studies yet.

Generally, there are different (epi)genetic pathways to CRC development, and the cancers resulting from each pathway have specific molecular characteristics that often associated with distinct prognosis trajectories. Therefore, it is also likely that these cancers have a distinct etiology. Diet and lifestyle factors may not only play a role in causing mutations and epigenetic changes, but also in enhancing tumor growth in tissues that have already acquired specific (epi) genetic aberrations. There may be direct causal associations between diet and lifestyle factors and molecular changes in CRC, and establishing this is important for prevention strategies, and increasing the ability to better predict disease progression and prognosis.

Traditionally, epidemiological research has been used to investigate how an exposure may increase or decrease the risk of developing cancer, and pathological research has been used to explore molecular characteristics of tumors to predict prognosis and response to treatment. By combining these two disciplines, a relatively new field of scientific investigation has emerged: molecular pathological epidemiology (MPE) [10]. In this review, we describe the (epi)genetic molecular pathways leading to CRC; identify MPE studies from around the world that have studied molecular markers of these pathways in relation to diet and/or lifestyle factors; summarize the data published on such associations; and explore future perspectives in this realm of research. We focus on diet and lifestyle factors for which there is evidence for an association with CRC as identified by the World Cancer Research Fund reports. In addition, we review promising tumor markers and hypotheses, that warrant consideration in future studies.

Studies on the importance of diet and lifestyle factors for CRC survival according to molecular subtype of CRC are not reviewed due to the current paucity of data. In addition, studies focused on downstream expression of genes in CRC as outcome are not reviewed.

## (Epi)genetic Pathways to CRC

Although each individual CRC tumor is (epi) genetically complex, and arises and behaves in a unique manner, it is common to classify tumors according to a limited number of phenotypes, because it is assumed that tumors with similar molecular characteristics have arisen through common mechanisms [10].

There are two morphologic, multi-step pathways to CRC (the traditional adenoma-carcinoma pathway and the serrated neoplasia pathway), which are driven by three molecular carcinogenesis pathways (chromosomal instability (CIN), microsatellite instability (MSI), and epigenetic instability (primarily the CpG island methylator phenotype (CIMP)) [11•]. It is important to understand these pathways, because MPE studies have been used to identify disease subtypes that may benefit from certain behavioral interventions, and may be used to validate molecular markers for risk assessment, early detection, prognosis, and prediction [12••, 13].

### The Traditional Adenoma-Carcinoma Pathway

Tumors arising via the traditional adenoma-carcinoma pathway begin as premalignant lesions comprising of conventional, tubular or tubulovillous adenomas [11•], and account for approximately 60–90% of sporadic CRCs [2]. They are characterized by CIN, which describes a condition of aneuploidy that is caused by an accelerated rate of gains and losses of entire or large portions of the chromosome during cell division [14, 15]. CIN is associated with inactivating mutations or losses in the Adenomatous Polyposis Coli (*APC*) tumor suppressor gene, which occurs as an early event in this sequence [16]. Mutations in the *KRAS* oncogene, as well as *TP53*, *SMAD4*, and *PIK3CA* genes are also frequently observed [2]. With CIN, there is an increased rate of heterozygosity, which may contribute to the inactivation of tumor suppressor genes or activation of tumor oncogenes [17]. Descriptively, tumors that arise from this pathway are more often associated with male sex, and observed in the distal colon [11•].

### Serrated Neoplasia Pathway

Approximately 10–30% of sporadic CRC tumors arise via the serrated neoplasia pathway [11•] and have distinctly different histology compared to tumors derived from the traditional adenoma-carcinoma sequence. They are characterized by MSI, a form of genetic instability characterized by length alterations within simple repeated microsatellite sequences of DNA. This is the result of strand slippage during DNA replication, which is not repaired due to a defective postreplication mismatch repair system [18]. An early event of these tumors is mutation of the *BRAF* proto-oncogene, which inhibits normal apoptosis of colonic epithelial cells [19]. The driving force of the serrated neoplasia pathway is the CpG methylator phenotype (CIMP), a form of epigenetic instability responsible for silencing a range of tumor suppressor genes, including *MLH1* [2]*.* Loss of *MLH1* is thought to cause microsatellite instability (MSI) and once *MLH1* is inactivated, the rate of progression to malignant transformation is rapid [19]. Descriptively, these tumors are more frequently associated with female sex, and are observed in the proximal colon [11•].

### Insights from the Cancer Genome Atlas Study

The Cancer Genome Atlas study, a collaboration between the National Cancer Institute (NCI) and the National Human Genome Research Institute (NHGRI), has generated a comprehensive, multi-dimensional map of the key genomic changes in CRC [20]. As recently summarized by Bae et al. [11•], the Cancer Genome Atlas study reports that CIN and MSI are mutually exclusive. CIMP, on the other hand, overlaps with the MSI pathway because of sporadic MSI-high CRCs, which are also usually CIMP-high, but does not appear to be in an exclusive relationship with the CIN pathway [11•, 20]. CIMP-high tumors can exist in the absence of MSI-high, and these tumors show some copy number variations across the genome, but the degree of CIN is less pronounced than CIMP-negative, MSI-low tumors. This suggests that CIMP alone may not be enough for the malignant transformation of serrated polyps, and requires collaboration with either CIN or MSI to promote successful malignant transformation [11•, 20].

In an MPE paradigm, a potential etiological factor, such as diet or lifestyle, is assessed with risk of an outcome across strata of molecular characteristics for the disease of interest [12••]. For purposes of this review, focus is on MPE studies that have considered diet and lifestyle factors in conjunction with primary molecular markers of (epi)genetic instability. For the traditional adenoma-carcinoma pathway, these include CIN, *APC* mutation, *KRAS* mutation, and *TP53* mutation. For the serrated neoplasia pathway, these include *BRAF* mutation, MSI, hypermethylation of *MLH1*, and CIMP.

## MPE Studies on Diet, Lifestyle, and CRC

Because MPE is an emerging research field, studies are usually drawn from existing cohort and case-control studies that have collected pathology specimens [12••]. In the realm of CRC, it is not uncommon for some large, long-running, population-based studies to have thousands of CRC cases. However, obtaining tumor blocks and subsequently phenotyping molecular characteristics in sample numbers large enough for meaningful statistical analysis requires a significant investment of both time and money. Therefore, while many epidemiological studies have investigated associations between diet, lifestyle, and CRC, the number of studies that have embarked on MPE investigations considering such associations is still currently quite limited.

### The Current Review

We reviewed the literature by searching combinations of key words (molecular pathological epidemiology, prospective cohort study, case-control study, *KRAS* mutation, *APC* mutation, Microsatellite Instability, CpG Island Methylator Phenotype, CIMP, *BRAF* mutation) in Pubmed and EMBASE databases, as well as by analyzing proceedings and participants of the International Molecular Pathological Epidemiology Meeting Series. Eight prospective cohort studies, five case-control studies, and one cross-sectional study that explicitly presented data on molecular markers of (epi)genetic instability were identified (Table 1). However, one cohort study did not further consider associations with diet and lifestyle factors [71], so for purposes of this review, was excluded from discussion. Of the remaining studies, associations have been published on molecular endpoints of CRC in relation to smoking, alcohol consumption; body mass index (BMI); waist:hip ratio; adult attained height; physical activity; early life energy restriction; ethnicity; dietary acrylamide, fiber, fat, methyl donors, omega 3 fatty acids; meat intake, including total protein, processed meat, and heme iron; and vegetable intake. For purposes of comparison and discussion, statistical associations are summarized in Tables 2 and 3, according to markers of the traditional adenoma-carcinoma and serrated neoplasia pathways, respectively, and the impact of these findings on advancing knowledge of CRC etiology is described in further detail below.

**Table 1:** Epidemiological studies that have collected molecular data according to (epi)genetic characteristics of colorectal cancer

Study	Country	*N*	Tumor characteristics
Prospective cohort studies			
European Prospective Investigation into Cancer (EPIC) Norfolk [21–24]	England	30,441	*APC* mutation and promoter hypermethylation, *BRAF* mutation, *KRAS* mutation, *MLH1* promoter hypermethylation, *TP53* mutation
Iowa Women’s Health Study (IWHS) [25–29]	USA	41,836	*BRAF* mutation, CIMP, *KRAS* mutation, MSI
Health Professionals Follow-up Study [10, 30–37]	USA	173,229	*BRAF* mutation, CIMP, KRAS *mutation*, LINE-1 hypomethylation, MSI, *PIK3CA* mutation
Malmo Diet and Cancer Study (MDCS) [26]	Sweden	29,098	*BRAF* mutation, *KRAS* mutation, MSI
Melbourne Collaborative Cohort Study (MCCS) [38, 39•, 40]	Australia	41,328	*BRAF* mutation, CIMP, MSI
Netherlands Cohort Study on Diet and Cancer (NLCS) [39•, 41–50, 51•, 52–55]	Netherlands	120,852	*APC* mutation*,* CIMP, CIN, *BRAF* mutation, *KRAS* mutation, *MGMT* promotor hypermethylation, *MLH1* promoter hypermethylation, MSI,
Nurses Health Study (NHS) [10, 30–37, 56, 57]	USA	77,443	*BRAF* mutation, CIMP, KRAS *mutation*, LINE-1 hypomethylation, MSI, *PIK3CA* mutation
Swedish Health and Disease Study (SHDS) [58]^1^	Sweden	166,414	CIMP, MSI
Case-control studies			
Colorectal Cancer: Chances for Prevention Through Screening (DACHS) [59]	Germany	1215 cases/ 1891 controls	MSI
Kaiser Permanente Medical Care Program of Northern California (KPMCP) and the state of Utah/Minnesota [60–64]	USA	1510 cases/ 2410 controls	*APC* mutation, *BRAF* mutation, CIMP, *KRAS* mutation, MSI, *TP53* mutation
Colon Cancer Family Registry (CCFR) [65]	USA	2253 cases/ 4486 controls	MSI
Dutch case-control study [66–68]	Netherlands	278 cases/ 414 controls	*MLH1* promoter hypermethylation, MSI, *APC* mutation,
Majorca case-control study [69]	Spain	286 cases/295 controls	*KRAS* mutation
Cross-sectional studies			
Martinez et al. [70]	Spain	623	*APC* mutation, *KRAS* mutation

One study did not publish data on these molecular endpoints with respect to diet and lifestyle factors

**Table 2:** Associations between diet and lifestyle factors and markers of the traditional adenoma-carcinoma pathway to CRC

				*APC* mutation	APC wildtype	*KRAS* mutation	KRAS wildtype	*TP53* mutation^6^	*TP53* wildtype
Exposure	Classification of exposure	Sex	*N*						
Prospective cohort studies				HR (95%CI)	HR (95%CI)	HR (95%CI)	HR (95%CI)	HR (95%CI)	HR (95%CI)
Smoking									
Smoking status									
Weijenberg et al. NLCS^1^ [42]	ex-smoker vs. never smoker	total	648			1.15 (0.79–1.66)	1.26 (0.96–1.66)		
Samadder et al. IWHS^2^ [25]	ever smoker vs. never smoker	women	505			1.05 (0.74–1.50)	1.23 (0.97–1.57)		
Age at smoking initiation									
Samadder et al. IWHS [25]	< 30 years vs never smoker	women	505			1.01 (0.70–1.46)	1.35 (1.06–1.72)		
Smoking duration									
Luchtenborg et al. NLCS [41]	> = 50 years vs. never smoker	total	661	1.15 (0.56, 2.37)	1.47 (0.84–2.56)				
Samadder et al. IWHS [25]	> = 40 years vs. never smoker	women	505			1.09 (0.65–1.83)	1.40 (0.99–1.97)		
Cumulative pack years									
Samadder et al. IWHS [25]	> = 40 years vs. never smoker	women	505			0.72 (0.36–1.44)	1.55 (1.07–2.25)		
Alcohol consumption									
Bongaerts et al. NLCS [43]	> 30 g/day vs. abstaining	total	578			1.13 (0.7–1.9)	N/A		
Gay et al. EPIC-Norfolk^3^ [22]	g/day; per 1SD increase	total	185	1.63 (1.13–2.35)	N/A				
Jayasakara et al. MCCS^4^ [38]	per 10 g/day increment	total	922			1.07 (1.00–1.15)	1.03 (0.98–1.08)		
Indicators of energy balance									
Body mass index									
Branstedt et al. Malmo diet and cancer study [26]	kg/m2; highest vs. lowest quartile	men	280					1.69 (0.99–2.82)	1.44(0.90–2.30)
		women	304					1.65 (0.95–2.89)	1.61(0.96–2.71)
Waist-hip ratio									
Branstedt et al. Malmo diet and cancer study [26]	cm; highest vs. lowest quartile	men	280					1.72 (1.02–2.91)	1.52(0.93–2.47)
		women	304					1.41 (0.87–2.31)	1.48(0.88–2.48)
Height									
Branstedt et al. Malmo diet and cancer study [26]	cm; highest vs. lowest quartile	men	280					1.65(0.93–2.92)	1.13(0.68–1.87)
		women	304					0.78(0.43–1.39)	2.17(1.25–3.76)
Dietary fiber									
Gay et al. EPIC- Norfolk [22]	g/day; +1SD increase	total	185	1.03 (0.75–1.43)	N/A				
Dietary Fat									
Brink et al. NLCS [48]	g/day PUFA (+1 SD)	total *colon rectum*	476			1.21(1.05–1.41)	0.94 (0.83--1.07)		
			176			0.99 (0.77–1.24)	0.97 (0.78–1.21)		
	g/day Linoleic Acid (+1 SD)	*colon rectum*	476			1.22 (1.05–1.42)	0.97 (0.86–1.10)		
			176			1.00 (0.77–1.29)	0.99 (0.80–1.23)		
Weijenberg et al. NLCS [69]^5^	g/day Linoleic Acid (+1 SD)	total *colon*	428			1.41 (1.18–1.69)	0.98 (0.84–1.15)		
Dietary methyl donors									
Folate									
de Vogel et al. NLCS [50]	micrograms/day; highest vs. lowest tertile	*colon* men women	213	2.77(1.29–5.95)	0.58 0.32–1.05				
			186	0.91(0.27–3.06)	0.93				
		*rectum* men women	84	0.92 (0.29–2.99)	(0.31–2.72)				
			45	1.25 (0.25–	1.80 (0.46–6.98)				
Dietary meat									
Total protein									
Gay et al. EPIC-Norfolk [22]	g/day; per 1 SD increase	total	185	1.21 (0.84–1.75)	N/A				
Red meat									
Gay et al. EPIC-Norfolk [22]	g/day; per 1 SD increase	total	185	1.17 (0.85–1.59)	N/A				
Processed meat									
Gay et al. EPIC-Norfolk [22]	g/day; per 1 SD increase	total	185	1.25 (0.91–1.72)	N/A				
Dietary heme iron									
Gay et al. EPIC-Norfolk [22]	mg/day; per 1 SD increase	total	185	1.50 (1.09–2.09)	N/A				
Gilsing et al. NLCS [51•]	mg/day; highest vs. lowest tertile	total	675	1.22 (0.79–1.89)	1.40 (1.06–1.84)	1.73 (1.08–2.77)	1.33 (0.99–1.77)	1.58(1.10–2.27)	1.15 (0.75–1.76)
				*APC* mutation	APC wildtype	*KRAS* mutation	KRAS wildtype	*TP53* mutation	*TP53* wildtype
CASE-control studies				OR (95% CI)	OR (95% CI)	OR (95% CI)	OR (95% CI)	OR (95% CI)	OR (95% CI)
Smoking									
Diergaarde et al. [72]	never vs. ever smoker	total	176 cases/249 controls	0.7 (0.4–1.4)	1.2 (0.7–2.1)	1.4 (0.7–2.8)	0.8 (0.5–1.4)	0.9 (0.4–1.9)	1.0 (0.6–1.7)
Curtin et al. 2009 [60]	> 20 pack years vs. non-smokers	*rectal* total	750 cases/ 1201 controls			1.3 (0.9–1.9)	N/A	1.4 (1.02–2.0)	N/A
Alcohol									
Diergaarde et al. [68]	highest vs. lowest tertile	*colon* total	184 cases/254 controls	0.5 (0.3–1.1)	1.7 (1.0–3.0)				
Dietary vegetable intake									
Diergaarde et al. [68]	highest vs. lowest tertile	*colon* total	184 cases/254 controls	0.6 (0.3–1.3)	0.3 (0.2–0.5)				
Dietary meat intake									
Diergaarde et al. [68]	highest vs. lowest tertile	*colon* total	184 cases/254 controls	1.7 (0.8–3.6)	1.5 (0.7–3.0)				
Dietary fish intake									
Diergaarde et al. [68]	highest vs. lowest tertile	*colon* total	184 cases/254 controls	1.4 (0.7–2.8)	0.9 (0.5–1.6)				
Dietary fat intake									
Diergaarde et al. [68]	highest vs. lowest tertile	*colon* total	184 cases/254 controls	4.5 (1.6–12.8)	1.6 (0.7–3.3)				
Cross-Sectional Studies				OR (95% CI)					
Smoking									
Martinez et al. [70]	smoker vs. never smoker	men	623	5.6 (1.6–20.4)					

^1^Netherlands Cohort Study on diet and cancer

^2^Iowa Women’s Health Study

^3^European Prospective Investigation into Cancer, Norfolk

^4^Melbourne Collaborative Cohort Study

^5^Activating mutations only

^6^Most presented studies on *TP53* are based on expression data except for those from Curtin et al. [60] which is based on mutation data. Nevertheless, results are provided because these studies also included other relevant end-points in this table or in Table 3

**Table 3:** Associations between diet and lifestyle factors and markers of the serrated neoplasia pathway to CRC

				*BRAF* mutation+	BRAF wildtype	CIMP+	CIMP-0	*MLH1* promoter hyper-methylation	*MLH1* normal	MSI +	MSS
Exposure	Classification of exposure	Sex	N								
Prospective cohort studies				HR (95%CI)	HR (95%CI)	HR (95%CI)	HR (95%CI)	HR (95%CI)	HR (95%CI)	HR (95%CI)	HR (95%CI)
Smoking*											
smoking status											
Limsui et al. IWHS^1^ [58]	ever smoker vs. never smoker	total	555	1.92 (1.22–3.02)	0.91 (0.65–1.27)	1.88 (1.22–2.90)	0.91 (0.64–1.29)			1.99 (1.26–3.14)	0.94 (0.68–1.31)
Nishihara et al. NHS^2^ [30]	current smoker vs. never smoker	total	1260	1.22 (0.98–1.52)	1.22 (0.98–1.52)	2.08 (1.35–3.20)	1.12 (0.89–1.41)			2.05 (1.29–3.26)	1.14 (0.91–1.42)
Age at smoking initiation											
Limsui et al. IWHS [58]	< 30 years vs never smoker	total	555	1.64 (1.14–2.35)	1.05 (0.83–1.33)	1.53 (1.08–2.17)	1.08 (0.85–1.38)			1.69 (1.17–2.44)	1.06 (0.84–1.34)
Nishihara et al. NHS [30]	< 20 years vs never smoker	total	1260	1.20 (0.83–1.72)	1.12 (0.97–1.31)	1.44 (1.02–2.01)	1.11 (0.95–1.30)			1.39 (0.97–1.99)	1.10 (0.94–1.28)
Smoking duration											
Limsui et al. IWHS [58]	> = 40 years vs. never smoker	total	555	1.58 (0.95–2.62)	1.07 (0.76–1.50)	1.69 (1.05–2.70)	1.00 (0.70–1.45)			1.72 (1.04–2.85)	1.06 (0.75–1.49)
Cumulative pack years											
Limsui et al. IWHS [58]	> = 40 years vs. never smoker	total	555	1.87 (1.09–3.21)	1.04 (0.71–1.53)	1.77 (1.05–2.99)	1.11 (0.75–1.64)			1.86 (1.06–3.24)	1.06 (0.72–1.55)
Nishihara et al. NHS [30]	> = 40 years vs. never smoker	total	1260	2.0 (1.37–2.92)	1.18 (0.98–1.43)	2.12 (1.48–3.03)	1.14 (0.94–1.39)			2.27 (1.56–3.31)	1.15 (0.95–1.39)
Alcohol consumption											
Bongaerts et al. NLCS^3^ [44]	> 30 g/day vs. abstaining	total	573							1.59 (0.4–5.8)	1.15 (0.5–2.7)
Gay et al. EPIC-Norfolk^4^ [22]	g/day; per 1SD increase	total	185								
Razzak et al. IWHS [28]	> 30 g/day vs. abstaining	women	732	0.73 (0.25–2.08)		0.53(0.16–1.74)				0.75 (0.26–2.16)	
Jayasakara et al. MCCS^5^ [38]	per 10 g/day increment	total	922	0.89 (0.78–1.01)	1.06 (1.01–1.11)						
Indicators of energy balance											
Early life energy restriction											
Hughes et al. NLCS [46]	exposure to famine vs. no exposure	total	603			0.65 (0.45–0.92)	0.91 (0.73–1.23)			0.85 (0.53–1.37)	0.84 (0.69–1.03)
Body mass index											
Hughes et al. NLCS [45]	highest vs. lowest quartile	total	603			1.45 (0.90–2.35)	1.03 (0.69–1.54)				
Hughes et al. NLCS/MCCS [39•]	highest vs. lowest quartile	total	1460	1.04 (0.69–1.58)	1.38 (1.15–1.66)					1.11 (0.70–1.76)	1.33 (1.11–1.60)
Branstedt et al. Malmo diet and cancer study [26]	highest vs. lowest quartile	men	280							2.47 (0.84–7.26)	1.37(0.95–1.99)
		women	304							0.91(0.39–2.25)	1.90(1.23–2.93)
Waist-hip ratio											
Hughes et al. NLCS [45]	highest vs. lowest quartile of skirt/trouser size;	total	603			1.90 (0.86–4.15)	1.39 (0.87–2.23)				
	per 2 skirt/trouser sizes					1.20 (1.01–1.43)	1.15 (1.04–1.28)				
Hughes et al. NLCS/MCCS [39•]	highest vs. lowest quartile of waist measurement	total	1460	1.40 (0.92–2.13)	1.38 (1.15–1.66)					1.40 (0.87–2.24)	1.60 (1.33–1.91)
Branstedt et al. Malmo diet and cancer study [26]	cm waist:hips;	men	280							1.52 (0.48–4.80)	1.36 (0.93–1.98)
	highest vs. lowest quartile	women	304							0.96 (0.41–2.27)	1.10 (0.76–1.60)
Height											
Hughes et al. NLCS/MCCS [39•]	per 5 cm increase	total	1460	1.23 (1.11–1.37)	1.08 (1.03–1.13)					1.26 (1.13–1.40)	1.08 (1.03–1.14)
	highest vs. lowest quintile			1.87 (1.26–2.77)	1.31 (1.09–1.56)					2.18 (1.38–2.44)	1.35 (1.13–1.60)
Branstedt et al. Malmo diet and cancer study [26]	highest vs. lowest quartile	men	280							1.79(0.55–5.77)	1.25(0.83–1.87)
		women	304							1.43(0.61–3.38)	1.28(0.83–1.97)
Physical activity											
Hughes et al. NLCS [45]	intermediate vs. low level	total	603			0.50 (0.30–0.81)	0.81 (0.61–1.07)				
Dietary methyl donors											
Folate											
de Vogel et al. NLCS [52]		men	367	3.04 (1.13–8.20)	N/A			0.88 (0.36–2.14)	N/A	0.78 (0.23–2.67)	N/A
		women	281	1.42 (0.51–3.92)				0.88 (0.33–2.32)		0.72(0.19–2.72)	
de Vogel et al. NLCS [53]	highest vs. lowest tertile	total	609			0.83 (0.52–1.35)	1.05 (0.75–1.47)				
Schernhammer et al. NHS [56]	highest vs. lowest quartile	women	387	0.80 (0.57–1.09)	0.89 (0.51–1.57)	0.98 (0.54–1.77)	0.73 (0.53–1.02)				
Vitamin B2											
de Vogel et al. NLCS [52]	highest vs. lowest tertile	men	367	0.79 (0.28–2.24)	N/A			0.93 (0.35–2.46)	N/A	1.59 (0.56–4.53)	N/A
		women	281	0.93 (0.3–2.91)				0.94 (0.39–2.26)		1.26(0.37–4.23)	
de Vogel et al. NLCS [53]	highest vs. lowest tertile	total	609			1.16 (0.72–1.87)	0.97 (0.72–1.31)				
Vitamin B6											
de Vogel et al. NLCS [52]	highest vs. lowest tertile	men	367	1.04 (0.35–3.08)	N/A			3.23 (1.15–9.06)	N/A	1.82 (0.57–5.80)	N/A
		women	281	0.97 (0.39–2.46)				1.61 (0.70–3.71)		1.10 (0.36–3.39)	
de Vogel et al. NLCS [53]	highest vs. lowest tertile	total	609			1.13 (0.71–1.80)	1.33 (0.97–1.83)				
Schernhammer et al. NHS [56]	highest vs. lowest quintile	women	387	0.73 (0.46–1.16)	1.15 (0.58–2.28)	1.24 (0.61–2.52)	0.77 (0.48–1.23)				
Methionine											
de Vogel et al. NLCS [52]	highest vs. lowest tertile	men	367	0.28 (0.09–0.86)	N/A			0.42 (0.14–1.25)	N/A	0.35 (0.07–1.83)	N/A
		women	281	2.06 (0.67–6.32)				1.13 (0.39–2.29)		1.15 (0.33–4.01)	
de Vogel et al. NLCS [53]	highest vs. lowest tertile	total	609			0.80 (0.49–1.31)	0.81 (0.59–1.10)				
Schernhammer et al. NHS [56]	highest vs. lowest quintile	women	387	1.01 (0.71–1.45)	0.65 (0.35–1.20)	0.77 (0.41–1.42)	1.04 (0.73–1.49)				
Vitamin B12											
Schernhammer et al. NHS [56]	highest vs. lowest quintile	women	387	0.92 (0.65–1.28)	0.78 (0.42–1.48)	0.77 (0.40–1.49)	0.99 (0.70–1.39)				
Dietary marine omega-3											
Song et al. NHS [31]	≥ 0.30 g/d vs < 0.10 g/d	total	1125	0.47 (0.24–0.93)	0.90 (0.72–1.13)	0.62 (0.37–1.04)	0.93 (0.74–1.17)			0.54 (0.35–0.83)	0.97 (0.78–1.20)
Case-control studies				OR (95% CI)	OR (95% CI)	OR (95% CI)	OR (95% CI)	OR (95% CI)	OR (95% CI)	OR (95% CI)	OR (95% CI)
Smoking											
Slattery et al. [62]	> 20 cigarettes a day vs. no smoking	*colon* men	821 cases/ 1283 controls							1.6 (1.0–2.5)	
		women	689 cases/ 1111 controls							2.2 (1.4–3.5)	
Samowitz et al. [61]	> 20 cigarettes a day vs. no smoking	*colon* total	1315 cases/ 2392 controls	3.16 (1.80–5.54)		2.06 (1.43–2.97)				with BRAF+: 3.00 (1.42–6.37) with CIMP+: 2.36 (1.30–4.29)	
Curtin et al. [60]	> 20 pack years vs. non-smokers	*colon* total	750 cases/ 1201 controls	4.2 (1.3–14.2)		1.5 (0.8–2.8)				5.7 (1.1–29.8)	
Poynter et al. [65]	> 30 pack years vs. non smokers	*total*	2253 cases/ 4486 controls							1.94 (1.09–3.46)	
Alcohol consumption											
Slattery et al. [63]	long term alcohol consumption	total	1510 cases/ 2410 controls							1.6 (1.0–2.5)	
Poynter et al. [65]	> 12 drinks per week vs. none	total	2253 cases/ 4486 controls							0.63 (0.35–1.13)	
Diergaarde et al. [67]	highest vs. lowest tertile	*colon* total	184 cases/254 controls							1.9 (0.8–4.7)	1.0 (0.6–1.8)
Body mass index											
Slattery et al. [62]	kg/m2; highest tertile vs. lowest tertile	*colon* men	821 cases/ 1283 controls							0.5 (0.3–0.9)	
		women	689 cases/ 1111 controls							1.1 (0.7–1.7)	
Hoffmeister et al. [59]	per 5 kg/m2 increase		men 641 cases/ 1117 controls							1.22 (0.82–1.81)	
			women 459 cases/ 774 controls							2.04 (1.50–2.77)	
Physical activity											
Slattery et al. [62]	low vs. high	*colon* men	821 cases/ 1283 controls							1.3 (0.7–2.3)	
		women	689 cases/ 1111 controls							0.8 (0.5–1.2)	
Dietary fruit intake											
Diergaarde et al. [67]	highest vs. lowest tertile	*colon* total	184 cases/254 controls							0.6 (0.2–1.4)	0.8 (0.5–1.3)
Dietary meat intake											
Diergaarde et al. [67]	highest vs. lowest tertile	*colon* total	184 cases/254 controls							0.5 (0.2–2.6)	1.5 (0.9–2.6)
Dietary vegetable intake Diergaarde et al. [67]	highest vs. lowest tertile	*colon* total	184 cases/254 controls							0.4 (0.1–0.9)	0.4 (0.2–0.7)

*Luchtenborg et al. 2005: daily number of cigarettes was associated with a dose-response in MLH1 normal cases, although case numbers were small

^1^Iowa Women’s Health Study

^2^Nurses Health Study/Health Professional’s Follow-up Study

^3^Netherlands Cohort Study on diet and cancer

^4^European Prospective Investigation into Cancer- Norfolk

^5^Melbourne Collaborative Cohort Study

### Smoking

Smoking has been studied in relation to both the traditional adenoma-carcinoma pathway [25, 41, 42, 58, 70, 72] and the serrated neoplasia pathway [30, 58, 60–62, 65]. As described in the proceedings of the third international MPE meeting, smoking provides one of the best examples of how MPE research can better predict CRC compared to epidemiological studies without molecular classification [12••]. Meta-analysis of traditional epidemiological studies showed only a modest link between smoking and CRC (i.e., a RR usually below 1.2) [73], which may lead one to believe that smoking is not a convincing risk factor for CRC. However, with the advent of MPE, it can be seen that once CRC cases are stratified by MSI or CIMP status, this risk increases up to two-fold for MSI-H and CIMP-H tumors in prospective cohort studies, while there are null associations for tumors not exhibiting these phenotypes (i.e., tumors of the traditional adenoma-carcinoma pathway). These data supports the premise that traditional epidemiological studies may mask true associations between some risk factors and cancer, and that MPE studies can shed light on true patterns of association.

### Alcohol Intake

The association between alcohol intake and CRC has been studied separately by tumor markers related to the traditional carcinoma-adenoma pathway [21, 38, 43, 66] and the serrated neoplasia pathway [22, 38, 44, 63, 67]. Although considered by the WCRF as a convincing risk factor for CRC in menand women, MPE data is conflicting. Acetaldehyde in alcoholic beverages is a highly toxic substance that is carcinogenic to humans. In one of the earliest case-control studies considering alcohol in relation to risk of *APC* mutations, Diergaarde et al. found that alcohol intake only increased the risk of *APC* wildtype tumors [66]. In 2006, Bongaerts et al. concluded that alcohol was not associated with tumors harboring mutations in the *KRAS* gene [43]; however, in 2016, Jayasekra et al. concluded that alcohol intake is associated with an increased risk of *KRAS* mutated and *BRAF* wildtype/*KRAS* wildtype tumors originating via the traditional adenoma-carcinoma pathway but not with *BRAF* mutated tumors originating via the serrated pathway [38]. This is in contrast to case-control data from Slattery et al., who was the first to report that alcohol intake is associated with MSI [63]. Some reasons for these discrepancies may include heterogeneity between the way that alcohol intake was measured (i.e. lifetime exposure, highest vs. lowest intake, continuous intake), and the inability to consider men and women separately in data analysis due to limitations with sample size. Another layer of complexity in the association between alcohol and CRC risk is that there are susceptibility genes in relation to alcohol metabolism not accounted for in MPE studies. This may also explain some of the observed heterogeneity.

### Indicators of Energy Balance

Indicators of energy balance include lifestyle factors that play a role in the development of body growth and obesity. These include body mass index (BMI), waist and hip circumference, adult-attained height, caloric intake and physical activity. The majority of MPE research on these factors has been conducted with respect to markers of the serrated neoplasia pathway [26, 39•, 45, 46, 59, 62, 64]. Although associations with *APC*, *KRAS*, and CIN have not been directly considered, the fact that BMI and waist measurements are positively associated with *BRAF* mutations and *BRAF*-wildtype, MSI and microsatellite stable tumors, and CIMP-H and non-CIMP tumors, is in accordance with WCRF evidence showing that overweight is a strong risk factor for CRC in general.

On the other hand, studies on adult-attained height and early life energy restriction suggest that timing of exposure may be important for influencing CRC risk. Height is a marker of aggregated fetal and childhood experience, and can be considered a proxy measure for important nutritional exposures, which affect several hormonal and metabolic axes [3]. Like body weight, adult-attained height is also an established risk factor for CRC in general; however, observations tend to be stronger for tumors demonstrating *BRAF* mutation and MSI [39•, 45]. One study on early life energy restriction showed that exposure to famine during childhood and adolescence decreased the risk of developing a tumor characterized by CIMP [46]. Taken together, this suggests that early life exposures may influence risk of epigenetic instability and CRC risk through the serrated neoplasia pathway, but data are scarce and more research is needed in this area.

### Dietary Factors

Because the majority of MPE studies are derived from larger cohort and case-control studies that were designed to consider outcomes between diet and cancer, and therefore have validated food frequency questionnaires in place, it is not uncommon for multiple dietary exposures to be presented in the same publication.

Red meat intake was identified by the WCRF as a probable risk factor for CRC, and MPE research supports that this may especially be true for tumors of the traditional adenoma-carcinoma pathway; dietary heme intake shows stronger associations with *KRAS*.mutated tumors than *KRAS* wildtype tumors. It has been hypothesized that heme can enhance the endogenous formation of carcinogenic *N*-nitroso compounds [51•]. The study by Gilsing et al. is important because it is the first human observational study providing evidence, as expected, for an association between heme and tumors with specific point mutations [51•].

Similarly, the first observational study showing that dietary acrylamide might be associated with CRC with specific somatic mutations, such as G > C or G > T mutations, was recently published [47], which supports the a priori hypothesis that metabolites of acrylamide are human carcinogens.

With respect to dietary fat, a high intake of polyunsaturated fat, in particular linoleic acid, has also been linked to *KRAS* mutations [49]. Intriguingly, and in contrast, it was recently reported that high marine omega-3 polyunsaturated fatty acid intake is associated with lower risk of MSI-high CRC but not MSS tumors, suggesting a potential role of omega-3 fatty acids in protection against CRC through DNA mismatch repair [31]. Calcium, milk, and garlic were not significantly associated with specific tumor subtypes in the reviewed publications [21, 22, 63, 64, 51•].

Alcohol is often considered in conjunction with dietary methyl donors such as folate, because folate may influence promoter methylation at gene promoters, and is depleted with alcohol intake. It has been hypothesized that methyl donors such as folate and methionine influence CRC through the serrated neoplasia pathway because of their role in methyl transport (i.e. a deficient status may result in a decrease in promotor hyper methylation, as observed in CIMP). Folate intake is associated with *BRAF* mutations, suggesting that it does play a role in epigenetic aberrations [52]. However, high folate consumption also appears to reduce the risk of *APC* wildtype colon tumors, while being positively associated with *APC* mutated colon tumors in men [50], indicating that folate may also enhance colorectal carcinogenesis through a distinct *APC* mutated pathway. More research, with attention to sample size, is needed to replicate and clarify these associations.

## Future Perspectives

In order to gain more insight into etiology and potential CRC interventions, it is important to continue investigating associations between diet, lifestyle factors and risk of different CRC subtypes. As mentioned previously, several studies have recently been publishing clustering CRC into specific subtypes [5•, 6, 8•, 9, 74]. The Cancer Genome Atlas study provides additional insights on how MPE studies in the realm of CRC should consider molecular markers and etiologic pathways [20].

As noted earlier, MPE studies are usually drawn from existing cohort and case-control studies. That means that in most cases, such studies have validated food-frequency and lifestyle questionnaires in place and in the future may have more tumor tissues available for molecular subtyping as cases continue to be identified. This will improve interpretation of research findings as One important limitations of MPE studies is limited sample size. Any molecular pathological epidemiology study conducted within a larger cohort will undergo multiple exclusions based on availability of tumor material and valid assay results. Therefore, the sample size for a study with molecular endpoints will always be smaller than the parent study. To analyze molecular data for associations with diet and lifestyle factors, a subset analysis for the different sub-sets is performed (i.e. CIMP-H vs CIMP-0; MSI-H vs MSS; *BRAF* mutated vs. *BRAF* wildtype tumors). The sample size for a subset, especially the rarer event (e.g., CIMP-H, MSI-H, *BRAF* mutated) may be too small to provide adequate statistical power, or limit the number of possible subtypes to be distinguished, even though this may at least in part be offset by more refined risk estimates in these subtypes.

Pooling data from independent studies may be a solution to this problem. To our knowledge, only one such MPE pooling data from the (NLCS) and the Melbourne Collaborative Cohort Study (MCCS) to assess the association between body size and CRC, by MSI and BRAF mutation, has been published so far. However, iin that study, pooling CIMP data was not possible due to methodological differences [39•]. This study highlights a unique challenge of pooling molecular data: it is important that similar definitions and laboratory analyses be used to define the phenotype in each study. We have previously published on the need for a global consensus on how to analyze and define CIMP [75, 76], but this is important for all molecular endpoints.

In a 2010 review on MPE of CRC, Ogino et al. identified that to overcome the unique challenges of this work, it would be necessary to coordinate research efforts around the world and to formulate a system where researchers could discover and validate new findings [4•]. Recently, The 3rd International Molecular Pathological Epidemiology (MPE) Meeting was held in Boston, which was attended by 150 scientists from 17 different countries [12••]. This meeting highlighted a new wave of research that is focused on increasing the understanding of the role that lifestyle/behavioral factors on modifying prognosis of diseases (including CRC) by considering specific disease subtypes. Such organization and collaboration will only expedite the creation of new, high quality studies, research questions, and answers around CRC etiology.

## Conclusion

Because CRC is a heterogeneous disease with several molecular subtypes, traditional epidemiological studies may mask completely or underestimate true associations between diet, lifestyle and disease risk. The WCRF has identified several convincing and probable risk factors for CRC, and by utilizing MPE can inform prevention and treatment strategies as well as predict prognosis for CRC.

MPE studies have also suggested that timing of exposure may be important for establishing patterns of epigenetic instability (e.g., as suggested by associations on adult-attained height and early life energy restriction with tumors exhibiting specific (epi)genetic markers). Furthermore, MPE studies offer the possibility to test hypotheses with regards to mutagenic effects (e.g., as suggested by the associations of heme iron and acrylamide with tumors exhibiting specific somatic mutations related to the exposure).

In the future, continuing collaboration and pooling data from high quality studies, including data on other molecular endpoints, may improve the strength of individual MPE findings, overcome the challenges of small sample sizes, and further pinpoint carcinogenic mechanisms leading to CRC.
